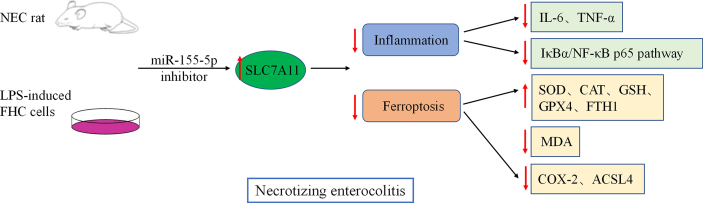# Silencing miR-155–5p expression improves intestinal damage through inhibiting inflammation and ferroptosis in necrotizing enterocolitis

**DOI:** 10.1016/j.heliyon.2024.e37087

**Published:** 2024-08-30

**Authors:** Le Zhang, Weilai Jin, Mengyuan Hu, Yinglin Su, Yiting Zhang, Fuqiang Yuan, Yuanyuan Fang, Zhengying Li, Yawen Li, Chaozhi Bu, Wenhao Zhou

**Affiliations:** a Key Laboratory of Birth Defects, Children's Hospital, Institutes of Biomedical Sciences, Fudan University, Shanghai, China; b Department of Neonatology, Affiliated Children's Hospital of Jiangnan University (Wuxi Children's Hospital), Wuxi, 214023, Jiangsu, China; c Department of Neonatology, The Affiliated Wuxi Children's Hospital of Nanjing Medical University, Wuxi, 214023, Jiangsu, China; d State Key Laboratory of Reproductive Medicine, Research Institute for Reproductive Health and Genetic Diseases, Wuxi Maternity and Child Health Care Hospital, Affiliated Women's Hospital of Jiangnan University, Wuxi, 214002, Jiangsu, China; e Guangzhou Women and Children's Medical Center, Guangzhou Medical University, Guangzhou, China

**Keywords:** MiR-155–5p, Ferroptosis, Inflammation, Necrotizing enterocolitis

## Abstract

**Background:**

Necrotizing enterocolitis (NEC) is a condition characterized by acquired damage to the mucosal lining, predominantly affecting premature infants. Bioinformatics assessments uncovered a notable rise in miR-155–5p expression in the intestinal tissues of infants suffering from NEC. Nevertheless, the development of NEC's underlying mechanisms and the role of miR-155–5p are still not well understood. This research aimed to explore the role of miR-155–5p in NEC and to elucidate its underlying mechanisms.

**Methods:**

To replicate NEC in vitro, lipopolysaccharide (LPS) was employed, whereas an in vivo rat model of NEC was established using formula feeding and exposure to hypoxia. Subsequently, levels of inflammatory cytokines, cell survival, and apoptosis rates were assessed. Various biochemical indicators such as glutathione (GSH), superoxide dismutase (SOD), catalase (CAT), and malondialdehyde (MDA) were measured utilizing a purchased diagnostic kit. For the assessment of reactive oxygen species (ROS) and mitochondrial membrane potential (MMP) within FHC cells, analysis by flow cytometry was conducted. Additionally, the technique of Western blotting was utilized to analyze the levels of ferroptosis-associated proteins. Moreover, hematoxylin and eosin (H&E) staining was carried out to observe the histopathological alterations in the intestinal tissue samples from rats with necrotizing enterocolitis (NEC).

**Results:**

Reducing miR-155–5p improved the survival of FHC cells exposed to LPS, decreased cell apoptosis, inflammation, and ferroptosis, and mitigated intestinal damage in NEC rats. Furthermore, SLC7A11 was found to be a direct target of miR-155–5p. The inhibition of miR-155–5p decreased LPS-induced inflammation and ferroptosis in both FHC cells and NEC rats by promoting SLC7A11 expression. This effect was evidenced by increased levels of ferroptosis-related proteins FTH1 and GPX4, decreased COX-2 and ACSL4 levels, lower lipid peroxidation marker MDA, reduced antioxidant markers GSH, SOD, and CAT, fewer IL-6 and TNF-α, and suppression of the IκBα/NF-κB p65 signaling pathway.

**Conclusions:**

In conclusion, reducing miR-155–5p could improve intestinal damage in NEC by inhibiting inflammation and ferroptosis. These findings may provide theoretical insights for the development of new therapies for NEC.

## Highlights

• Experimental NEC models were established in cell line and rat.
• Inhibition of miR-155–5p ameliorated intestinal injury of NEC via supressing ferroptosis process and inflammatory response.
• Our findings highlight a novel therapeutic target for NEC treatment.

## 1 Introduction

NEC is a kind of serious inflammatory condition of the intestines that impacts premature infants [1]. Risk factors such as pathogenic organisms, intestinal hypoxia/ischaemia and inflammation are thought to result in NEC [[2], [3], [4], [5]]. Moreover, NEC is marked by patchy ulceration, intense intestinal inflammation, and tissue necrosis [6]. At the same time, NEC is a major cause of death (15–30 %) among premature infants [7]. Regrettably, there is a shortage of targeted therapeutic approaches for NEC [8]. Hence, it is crucial to create innovative strategies for preventing and managing NEC.

MicroRNAs (miRNAs) are tiny, single-stranded noncoding RNAs, roughly 18–24 nucleotides long [[9], [10], [11]]. miRNAs regulate the gene expression at the posttranscriptional level [12,13]. Moreover, miRNAs are involved in various biological processes, such as cellular development, inflammation, and oxidative stress [[14], [15], [16]]. Growing evidence indicates that miRNAs are crucial in the development of NEC [17,18]. Zhu et al. discovered that reducing miR-34a could lessen intestinal damage and the inflammatory reaction in NEC rats [19]. Previous research indicated that blocking miR-124 could inhibit intestinal cell apoptosis in NEC rats [20]. Furthermore, it has reported that elevating miR-155 levels could promote experimental colitis in mice [21]. Shao et al. showed that miR-155 exhibited a proinflammatory function in the development of inflammatory bowel disease [22]. Nonetheless, the function of miR-155 in NEC progression is still not well understood.

Iron-mediated lipid peroxidation defines a novel mode of programmed cell demise termed ferroptosis [23,24]. Ferroptosis plays a significant role in the development of gut damage, including NEC [25]. SLC7A11 functions as a ferroptosis inhibitor by transporting extracellular cystine into cells, supporting GSH production, and removing surplus ROS from the body [26]. Inhibiting SLC7A11 leads to the blockade of GSH synthesis, impairs the antioxidant capacity of cells, and then leads to ferroptosis [27]. In this research, it was discovered that miR-155–5p is targeted directly by SLC7A11. As a result, we delved into the possibility that miR-155–5p could impact NEC by modulating SLC7A11. The findings from our study could provide valuable insights for subsequent research aimed at devising treatments that focus on targeting miR-155–5p.

## 2 Materials and methods

### 2.1 Data gathering and analysis of differential expression

To identify the differentially expressed miRNAs (DEmiRNAs) in intestinal tissues from NEC patient group compared to healthy control group, raw gene expression data were sourced from the GEO dataset (GSE68054). Statistical analysis of the DEGs was conducted using R software, with significance thresholds set at P < 0.05 and |log2 (FC)| > 2.

### 2.2 Cell culture and treatment

Human normal colorectal FHC cells (ATCC, originating from Maryland, United States) were maintained in a controlled environment at a temperature of 37 °C and in an atmosphere of 5 % carbon dioxide. The culture medium used was DMEM/F-12, sourced from Thermo Fisher Scientific, located in Waltham, United States, supplemented with 10 % serum derived from fetal bovines, provided by Gibco, based in Grand Island, United States. Additionally, the medium was fortified with 1 % penicillin-streptomycin, also from Gibco. To create a NEC cellular model, the FHC cells were subjected to a concentration of 50 μg per milliliter of lipopolysaccharide (LPS), obtained from Sigma Aldrich, headquartered in St Louis, United States, for a duration of 6 h.

### 2.3 Cell transfection

The regulatory miRNA inhibitors (inhibitor control), specific inhibitors targeting miR-124–5p, miR-132–3p, and miR-155–5p, as well as the miR-155–5p imitators and the imitator control were sourced from RiboBio Corporation (located in Guangzhou, China). For the introduction of nucleic acids into the cells, the Lipofectamine 2000 transfection agent (distributed by Sigma Aldrich, based in St. Louis, USA) was employed. Briefly, FHC cells (5 × 10^5^) were plated in 6-well plates with serum-free medium one day before transfection. On the following day, the miRNA inhibitors or mimics were diluted with Lipofectamine 2000 reagent in Opti-MEM medium (Gibco, Grand Island, US), mixed, and applied to the cells. Subsequent experiments were carried out 48 h after the transfection.

### 2.4 ELISA

The levels of IL-6 and TNF-α in cell supernatants and intestinal tissues were assessed using human (No. ELK1156) or rat (No. ELK1158) IL-6 ELISA Kits and human (No. ELK1190) or rat (No. ELK1396) TNF-α ELISA Kits. All these kits were provided by Beyotime Biotechnology (Beijing, China). The analysis was performed following the manufacturer's instructions.

### 2.5 Detection of oxidative stress

Furthermore, the levels of SOD, CAT, MDA, and GSH within the cell supernatant and the intestinal tissue were determined utilizing the SOD testing reagent set (Product Code: A001-3-2), the CAT testing reagent set (Product Code: A007-1-1), the MDA testing reagent set (Product Code: A003-1), and the total glutathione/reduced glutathione testing reagent set (Product Code: A061-1), following the producer's guidelines. The aforementioned assay kits were procured from the Nanjing Jiancheng Bioengineering Institute situated in Nanjing, PR China.

### 2.6 Determination of iron content

FHC cells were broken down with cell lysis buffer (Macklin, Shanghai, China), and subsequently, the iron content in the cell lysates was analyzed through colorimetric methods using an Iron Assay Kit (Sigma Aldrich, St Louis, US), according to the manufacturer's guidelines. The absorbance at 593 nm was recorded using a microplate reader (Tecan Infinite M200 pro, Switzerland).

In addition, the content of Fe^2+^ in FHC cells was observed by using the Phen Green™ SK (PGSK) probe. To encapsulate, FHC cultures in 24-well dishes underwent an initial fixation process with 4 % paraformaldehyde for a duration of half an hour. Following this, they were subjected to permeabilization for a further 10 min with a 0.1 % solution of Triton X-100. Post-permeabilization, the cells were incubated with the PGSK dye (manufactured by Beyotime, located in Beijing, China) for a 20-min interval, and the nuclei were counterstained with DAPI. The assessment of ferroptosis in these cells was conducted using a fluorescence microscope, model Olympus BX43, originating from Japan.

### 2.7 CCK-8 assay

Human FHC cells were planted at a concentration of 5000 cells per individual well in a 96-well culture dish and subjected to an overnight culture period. For each treatment scenario, there were six wells serving as duplicates. Subsequently, the cells were subjected to LPS treatment and co-transfected with either a control inhibitor or a miR-155–5p inhibitor as per the designated experimental protocols. At the 48-h mark, cell proliferation was assessed using the CCK-8 kit (Beyotime Institute, Beijing, China). Ten microliters of the CCK-8 reagent were introduced to each well and co-incubated with the cells for a further 2 h. The absorbance was recorded at 450 nm with a microplate spectrophotometer.

### 2.8 TUNEL assay

The evaluation of cell apoptosis was conducted in adherence to the protocol provided by the manufacturer for the In Situ Cell Death Detection Kit (produced by Roche Diagnostics, based in Mannheim, Germany). In essence, the FHC cells were subjected to a 60-min incubation period with the TUNEL reagent in a dark environment. Following this, the cell nuclei were dyed with DAPI (supplied by Beyotime, a company located in Beijing, China). To conclude the process, the identification of apoptotic cells was carried out using a fluorescence microscope.

### 2.9 Flow cytometry assay

For the assessment of ROS, FHC cell cultures were subjected to 2,7-dichlorofluorescin diacetate (provided by KeyGen Biotech Co., Ltd., situated in Nanjing, China) under dark conditions at a temperature of 37 °C for a duration of half an hour. Post-incubation, the cells underwent and were subsequently re-dispersed in phosphate-buffered saline. The determination of ROS concentrations was carried out with the aid of a flow cytometric instrument (manufactured by BD Biosciences, based in New Jersey, USA).

For MMP assessment, FHC cells were exposed to the JC-1 probe (Beyotime, Beijing, China) at 37 °C in the dark. After a 30-min incubation, a flow cytometer (BD Biosciences, New Jersey, US) was used to evaluate the fluorescence intensity.

### 2.10 Luciferase reporter assay

The Beyotime luciferase reporter construct, pGL6-miR, origin from Beijing, China, was employed to incorporate the SLC7A11 gene in either its mutated (MT) or natural (WT) state. Subsequently, these constructs were introduced into FHC cells alongside either miR-155–5p mimetics or a control mimic, via co-transfection with Lipofectamine 2000. Following a 48-h incubation period, the luciferase activity within the FHC cells was quantified using a dual-luciferase assay system, supplied by Promega, Madison, USA.

### 2.11 RT-qPCR assay

The entire RNA was isolated utilizing the TRIpure Total RNA Isolation Reagent (Aidlab, Beijing, China) and subsequently transformed into complementary DNA using the EntiLink™ First Strand cDNA Synthesis Kit. Quantitative polymerase chain reaction (qPCR) was performed with the EnTurbo™ SYBR Green PCR Master Mix to assess the expression levels of SLC7A11. β-actin served as the internal control for standardizing the expression of SLC7A11. All experimental reagents were sourced from ELK Biotechnology Co., Ltd. (Wuhan, China). Below are the sequences of the primers used: β-actin forward, 5′- GTCCACCGCAAATGCTTCTA-3′ and reverse, 5′- TGCTGTCACCTTCACCGTTC-3’; SLC7A11 forward, 5′- TGTGGGGTCCTGTCACTATTTG-3′ and reverse, 5′- GATATCACAGCAGTAGCTGCAGG-3’.

### 2.12 Western blot assay

The samples were subjected to electrophoresis on a 10 % polyacrylamide gel using SDS (30 μg per lane) before being electrophoretically transferred onto a PVDF membrane (manufactured by Millipore in Massachusetts, United States). Following this, the membrane underwent an overnight incubation at a temperature of 4 °C with specific primary antibodies directed against diverse protein targets: anti-SLC7A11 (ab302919, 1:1000 dilution, Abcam, UK), anti-COX-2 (12375-1-AP, 1:2000 dilution, Proteintech, US), anti-ACSL4 (ab205199, 1:1000 dilution, Abcam, UK), anti-FTH1 (DF6278, 1:1000 dilution, Affinity Biosciences, US), anti-GPX4 (DF6701, 1:1000 dilution, Affinity Biosciences, US), anti-p-IκBα (AF2002, 1:1000 dilution, Affinity Biosciences, US), anti-IκBα (AF5002, 1:1000 dilution, Affinity Biosciences, US), anti-p-p65 (ab76302, 1:1000 dilution, Abcam, UK), anti-p65 (80979-1-RR, 1:20000 dilution, Proteintech, US), and anti-β-actin (81115-1-RR, 1:20000 dilution, Proteintech, US). Following this, the membrane was subjected to the relevant secondary antibody for a duration of 60 min. Subsequently, the protein bands were identified with the ECL substrate (manufactured by Thermo Fisher Scientific, based in Waltham, USA) and the visualization process was carried out utilizing a chemiluminescent imaging device (model Alpha FluorChem Q, produced in the United States).

### 2.13 Animal study

The experimental Sprague‒Dawley (SD) rats were obtained from SPF (Beijing) Biotechnology Co., Ltd., with a total of 15 rats (6–8 g, 8 females and 7 males). The rat pups were divided into 3 groups (n = 5): the control, NEC, and NEC + miR-155–5p inhibitor groups. Every 5 h, formula gavage (Similac Advance infant formula: Esbilac canine milk replacer = 2:1; 50 μl/g) was administered to all animals in the NEC and NEC + miR-155–5p inhibitor groups, as previously described [28]. During the experiment, the rats underwent hypoxia (5 % O2, 95 % N2) twice daily for 10 min in a hypoxia chamber [28]. The rats in the control group were exposed via breastfeeding. Intraperitoneal injections of the miR-155–5p inhibitor were administered to the rats in the NEC + miR-155–5p inhibitor group. At the termination of the study on the fifth day, the subjects were humanely euthanized through neck manipulation. Subsequently, the gastrointestinal tissues were excised, preserved in wax, and divided into sections measuring 4 μm in thickness. The integrity of the gut tissues was evaluated through hematoxylin and eosin dye application. The procedures involving the animals were approved by the Laboratory Animal Ethics Committee of the Affiliated Wuxi Children's Hospital of Nanjing Medical University.

**Figure:**
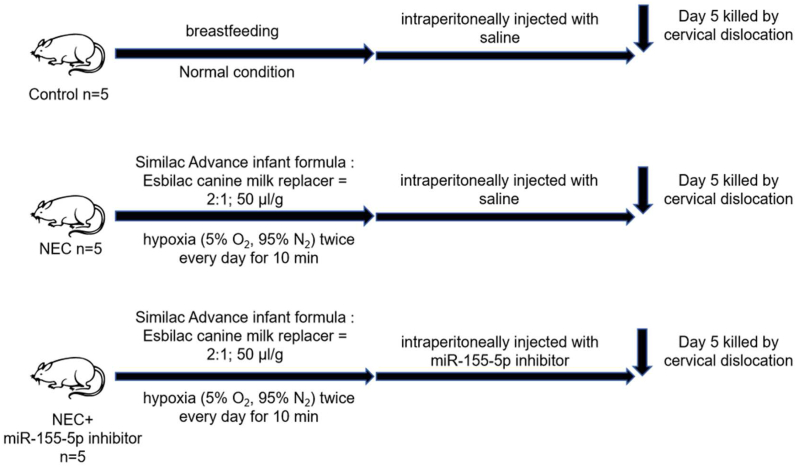

### 2.14 Statistical analysis

Data analysis was performed utilizing SPSS Statistics (Version 20, Chicago, IL, USA). The continuous data were averaged, and both addition and subtraction operations were applied. Additionally, the standard deviation was calculated. To examine the disparities between two groups, a *t*-test was executed. In cases of comparisons involving three or more groups, a one-way ANOVA and LSD test were used. The outcomes unveiled a noteworthy distinction between the two groups (P < 0.05).

## 3 Results

### 3.1 The reduction of miR-155–5p enhanced cell viability and reduced apoptosis, inflammation, and oxidative stress in FHC cells treated with LPS

In order to analyze the DEmiRNAs in intestinal tissues from NEC patients compared to healthy controls, data from the GSE68054 dataset in the GEO database were analyzed using R programming. As depicted in Fig. 1A and B, and Supplementary Fig. 1, a total of 52 DEmiRNAs were identified in the GSE68054 dataset. The expression levels of miR-124, miR-132, and miR-155 were significantly elevated in the intestinal tissue samples from patients with NEC when contrasted with those from individuals without the condition (Fig. 1A and B). Additionally, our research demonstrated that in LPS-treated FHC cells, miR-155–5p levels were increased, and its expression was markedly decreased by a miR-155–5p inhibitor (Fig. 2A). Diminishing the levels of miR-124–5p, miR-132–5p, or miR-155–5p significantly decreased the levels of IL-6 and TNF-α within FHC cells that were exposed to LPS (Fig. 2B and C). Since the miR-155 inhibitor exhibited the most pronounced anti-inflammatory impact in LPS-treated FHC cells, miR-155–5p was used for further exploration in our following study. We confirmed the elevated expression level of miR-155–5p in LPS-treated FHC cells using RT‒qPCR (Fig. 2C). Furthermore, LPS treatment markedly suppressed the viability of FHC cells and triggered cell apoptosis, however, these changes were restored by the miR-155–5p inhibitor (Fig. 2D and E). Moreover, oxidative stress in FHC cells was detected. LPS notably elevated the level of ROS (Fig. 3A), and increased the content of MDA (Fig. 3B), and decreased the levels of GSH, SOD and CAT (Fig. 3C–E) in FHC cells. However, the miR-155–5p inhibitor significantly reversed these effects. In summary, reducing miR-155–5p levels enhanced cell viability while decreasing apoptosis, inflammation, and oxidative stress in LPS-treated FHC cells.

**Fig. 1:**
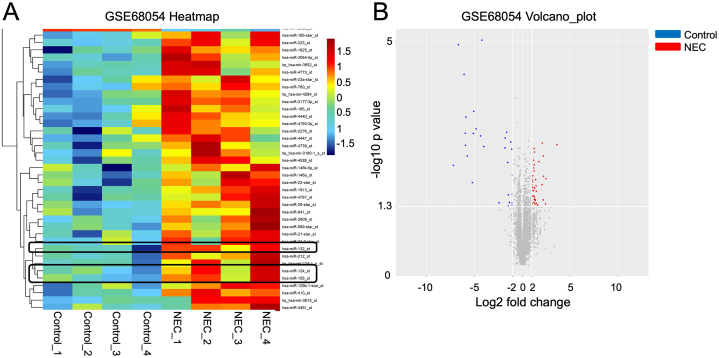
**Identification of DEmiRNAs in NEC. (A, B)** The DEmiRNAs in intestinal tissues between patients with NEC and healthy controls were presented in the heatmap and volcano plot.

**Fig. 2:**
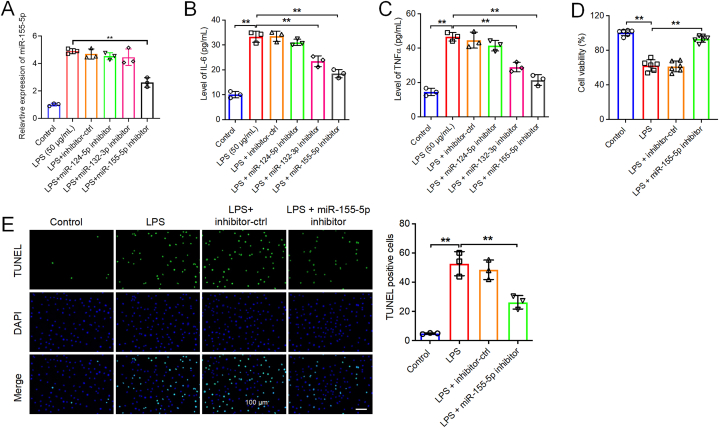
**Downregulation of miR-155-5p promoted the viability and suppressed the apoptosis and inflammation of LPS-treated FHC cells.** (A) FHC cells were stimulated with 50 μg/ml LPS for 6 h, and then transfected with inhibitor-ctrl, miR-124–5p inhibitor, miR-132–3p inhibitor or miR-155–5p inhibitor for 48 h. The levels of miR-155–5p in FHC cells were assessed by RT-qPCR. n = 3. (B and C) The level of IL-6 and TNF-α was evaluated by ELISA. n = 3. (D and E) FHC cells were stimulated with 50 μg/ml LPS for 6 h, and then transfected with inhibitor-ctrl or miR-155–5p inhibitor for 48 h. The viability and apoptosis of FHC cells were determined by CCK-8 (n = 6) and TUNEL (n = 3) assays respectively. Scale bar = 100 μm. **P < 0.01.

**Fig. 3:**
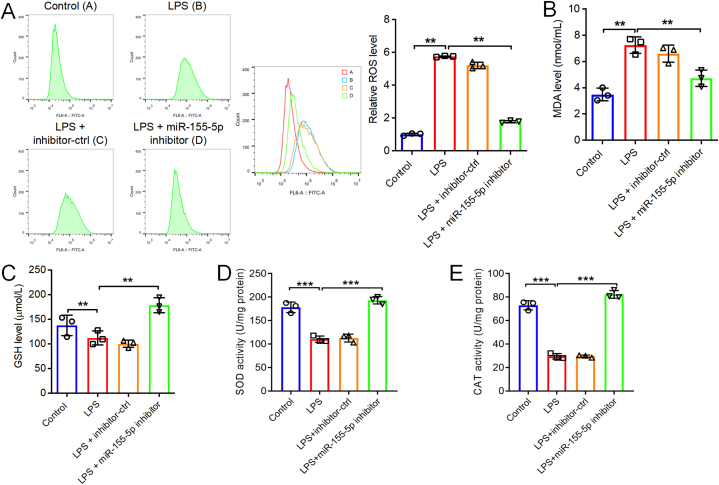
**Downregulation of miR-155-5p suppressed the oxidative stress of LPS-treated FHC cells.** FHC cells were stimulated with 50 μg/ml LPS for 6 h, and then transfected with inhibitor-ctrl or miR-155–5p inhibitor for 48 h. **(A)** ROS level in FHC cells were evaluated by flow cytometry. Levels of MDA **(B)**, GSH **(C)**, SOD **(D)** and CAT **(E)** in FHC cells were assessed by corresponding kit. n = 3, **P < 0.01, ***P < 0.001.

### 3.2 Downregulation of miR-155–5p suppressed mitochondrial damage and ferroptosis in LPS-treated FHC cells

**Fig. 4:**
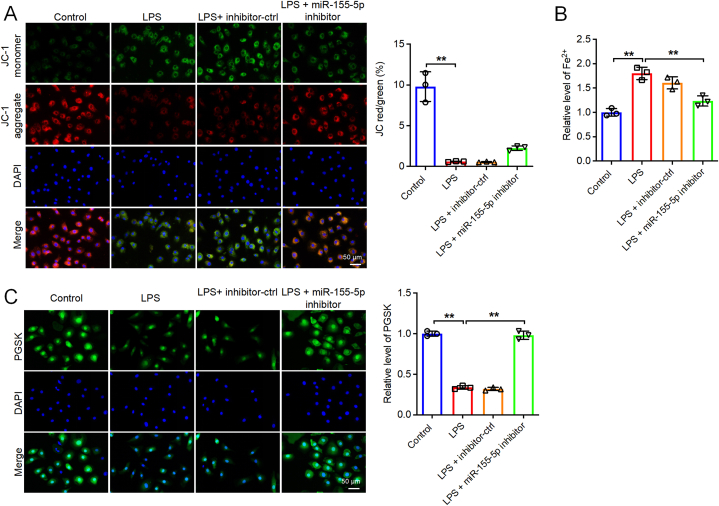
**Downregulation of miR-155-5p suppressed the mitochondrial damage and ferroptosis in LPS-treated FHC cells.** FHC cells were stimulated with 50 μg/ml LPS for 6 h, and then transfected with inhibitor-ctrl or miR-155–5p inhibitor for 48 h. **(A)** MMP in FHC cells was detected by JC-1 assay. **(B)** The iron levels in FHC cells were assessed by commercial kits. **(C)** The level of Fe^2+^ in FHC cells was detected by fluorescence intensity of PGSK. Scale bar = 50 μm. n = 3, **P < 0.01.

Here, we aimed to determine whether LPS treatment could induce ferroptosis in FHC cells and evaluate the effect of a miR-155–5p inhibitor on this process. The suppression of miR-155–5p effectively counteracted the decrease in mitochondrial membrane potential (MMP) triggered by LPS in FHC cellular models (Fig. 4A). Additionally, iron levels were significantly elevated in FHC cells exposed to LPS, but the miR-155–5p inhibitor mitigated this iron accumulation (Fig. 4B). The Fe2+ content in FHC cells was also measured using a PGSK probe. Fig. 4C demonstrates that the miR-155–5p inhibitor significantly restored the green fluorescence of PGSK in LPS-treated FHC cells. In summary, ferroptosis in FHC cells exposed to LPS was mitigated by reducing miR-155–5p.

### 3.3 Reduction of miR-155–5p prevents ferroptosis triggered by LPS in FHC cells by influencing SLC7A11 and impacting the IκBα/NF-κB p65 pathway

**Fig. 5:**
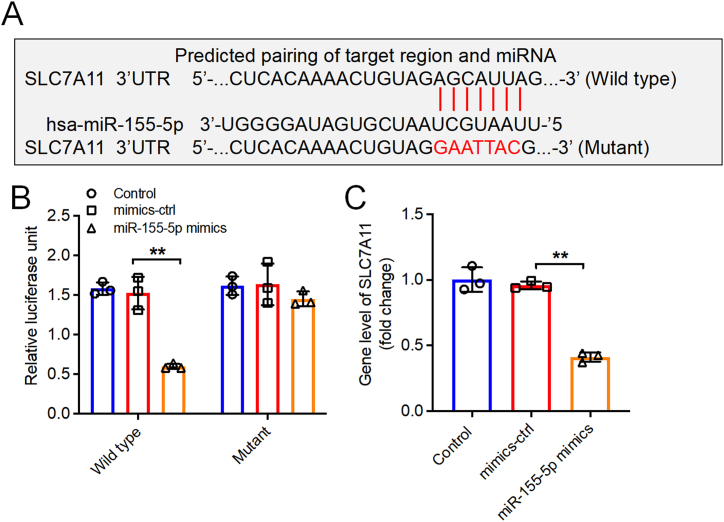
**SLC7A11 was a direct target of miR-155-5p. (A)** The putative binding sites of miR-155–5p on SLC7A11. **(B)** Luciferase assay of FHC cells co-transfected with SLC7A11-WT or SLC7A11-MT reporter plasmid and miR-155–5p mimic or mimics-ctrl. **(C)** The SLC7A11 level in FHC cells transfected with miR-155–5p mimics was assessed by RT-qPCR. n = 3, **P < 0.01.

Next, we sought to further investigate the mechanism through which miR-155–5p influences LPS-induced ferroptosis in FHC cells. We employed the TargetScan and miRwalk repositories to pinpoint prospective targets for miR-155–5p. Our investigation suggested that SLC7A11, a protein integral to the process of ferroptosis, could serve as a likely target for miR-155–5p (Fig. 5A). The incorporation of miR-155–5p mimics led to a notable decrease in luciferase expression in FHC cells when co-transfected with the wild-type SLC7A11, thereby validating SLC7A11 as a direct binding site for miR-155–5p (Fig. 5B). Additionally, overexpression of miR-155–5p markedly decreased the mRNA levels of SLC7A11 in FHC cells (Fig. 5C).

**Fig. 6:**
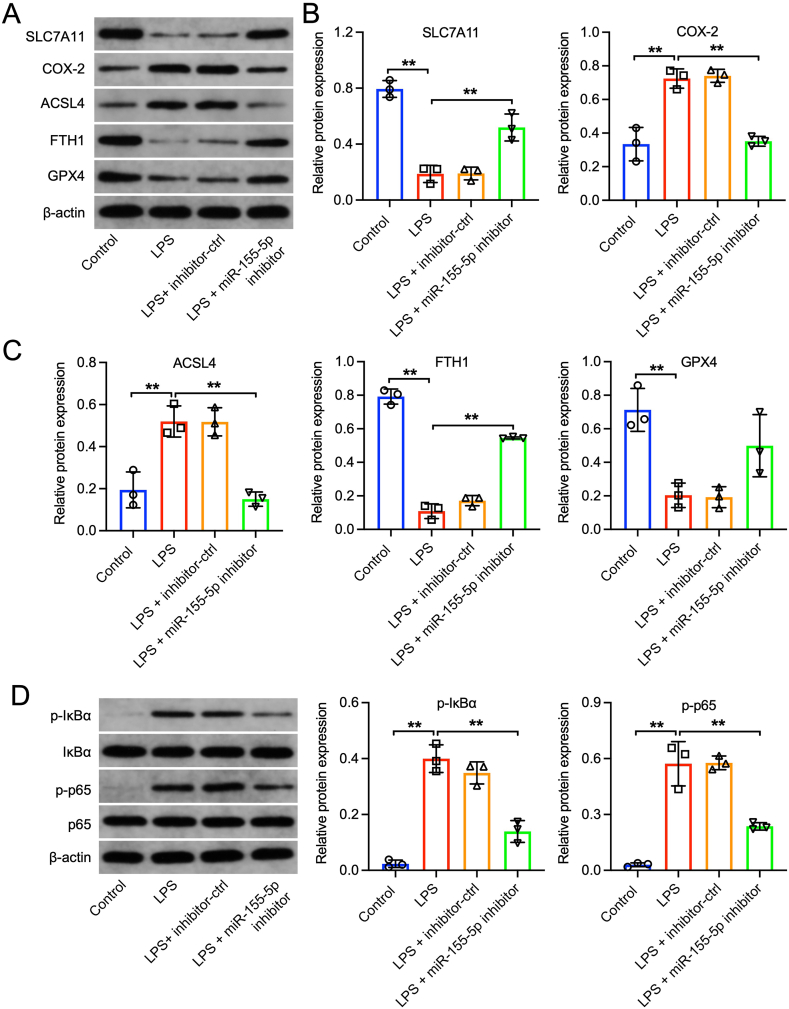
**Downregulation of miR-155-5p suppressed the ferroptosis in LPS-treated FHC cells via regulating SLC7A11 and IκBα/NF-κB p65 pathway.** FHC cells were stimulated with 50 μg/ml LPS for 6 h, and then transfected with inhibitor-ctrl or miR-155–5p inhibitor for 48 h (A, B, C) Western blot assay was applied to determine the expressions of SLC7A11, COX-2, ACSL4, FTH1, GPX4 in FHC cells. (D) Western blot assay was conducted to detect the expressions of p-IκBα, IκBα, p-p65 and p65 in FHC cells. n = 3, **P < 0.01.

Additionally, we assessed the protein levels of SLC7A11 and four ferroptosis-related factors (COX-2, ACSL4, FTH1, and GPX4). LPS treatment significantly reduced SLC7A11, FTH1, and GPX4 levels while increasing COX-2 and ACSL4 levels in FHC cells. The miR-155–5p inhibitor counteracted these effects (Fig. 6A, B, and 6C). We explored the potential of heightened miR-155–5p levels to mitigate inflammation through the suppression of the IκBα/NF-κB p65 signaling pathway's activation. Our findings revealed that the administration of the miR-155–5p inhibitor significantly curtailed the phosphorylation of IκBα and p65 instigated by LPS (Fig. 6D). In conclusion, reducing miR-155–5p effectively mitigated ferroptosis in LPS-treated FHC cells by promoting SLC7A11 expression.

### 3.4 Decreasing miR-155–5p alleviated intestinal damage, inflammation, and oxidative stress in NEC rats by modulating SLC7A11 and influencing the IκBα/NF-κB p65 signalling pathway

**Fig. 7:**
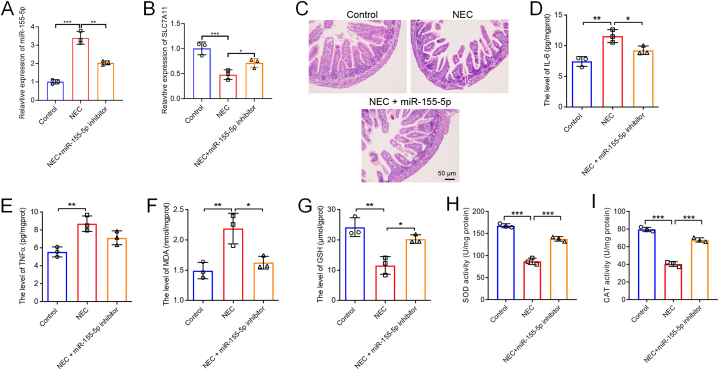
**Downregulation of miR-155-5p attenuated intestinal injury, inflammatory response, and oxidative stress in NEC rats.** (A) The relative expression of miR-155–5p in intestinal tissues of NEC rat and NEC + miR-155–5p inhibitor rat was evaluated by RT-qPCR. (B) The relative expression of SLC7A11 in intestinal tissues of NEC rat and NEC + miR-155–5p inhibitor rat was evaluated by RT-qPCR. (C) H&E staining showed the pathological changes in the intestine tissues. Scale bar = 50 μm. (D, E) The level of IL-6, TNF-α in intestinal tissues were assessed by ELISA. (F to I) Levels of MDA, GSH, SOD and CAT in intestinal tissues were assessed by corresponding kit. n = 3, *P < 0.05, **P < 0.01, ***P < 0.001.

**Fig. 8:**
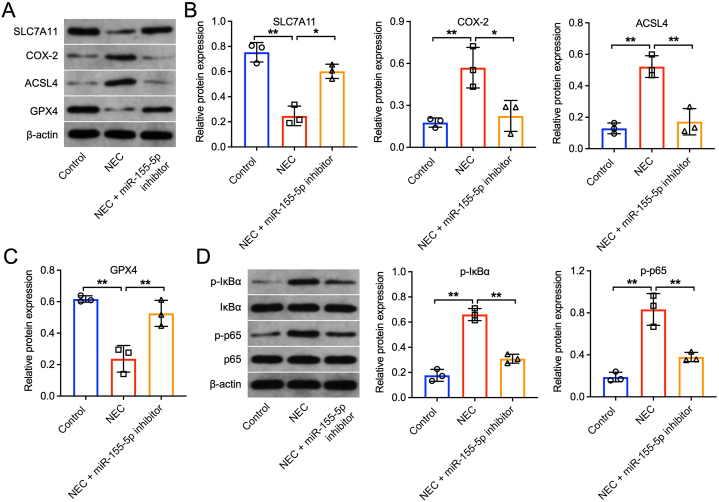
**Downregulation of miR-155-5p attenuated intestinal injury in NEC rats via modulating SLC7A11 affecting the IκBα/NF-κB p65 pathway.** (A, B, C) Western blot assay was applied to determine the expressions of SLC7A11, COX-2, ACSL4, GPX4 in intestine tissues. (D) Western blot assay was conducted to detect the expressions of p-IκBα, IκBα, p-p65 and p65 in intestine tissues. n = 3, *P < 0.05, **P < 0.01.

We then investigated the effects of reducing miR-155–5p in the NEC rat model. RT-qPCR first confirmed the elevated levels of miR-155–5p in the intestinal tissues of NEC rats and showed that the miR-155–5p inhibitor could lower this expression (Fig. 7A). Additionally, RT-qPCR results indicated a decline in the mRNA level of SLC7A11 in the NEC model group, with increased SLC7A11 levels observed upon inhibition of miR-155–5p expression (Fig. 7B). H&E staining demonstrated significant villi loss, crypt atrophy, and edema in the intestinal tissues of NEC rats, effects that were alleviated by the miR-155–5p inhibitor (Fig. 7C). Additionally, the miR-155–5p inhibitor notably reduced the levels of IL-6 and TNF-α in the intestinal tissues compared to the NEC group (Fig. 7D and E). The decrease of miR-155–5p also mitigated oxidative stress in these tissues. Specifically, the levels of MDA were significantly decreased, while GSH, SOD, and CAT levels were markedly increased in the NEC + miR-155–5p inhibitor group (Fig. 7F–I). In line with the in vitro results, miR-155–5p inhibition substantially elevated the expression of SLC7A11 and GPX4 while reducing COX-2 and ACSL4 levels in the intestinal tissues of NEC rats (Fig. 8A, B, and 8C). Furthermore, the miR-155–5p inhibitor greatly decreased the phosphorylation of IκBα and p65 in these tissues (Fig. 8D). Overall, lowering miR-155–5p improved intestinal damage, inflammation, and oxidative stress in NEC rats by boosting SLC7A11 expression.

## 4 Discussion

NEC is an acute inflammatory intestinal disease of premature neonates [29]. Evidence has shown that formula feeding, pathogenic organisms and intestinal hypoxia/ischaemia could lead to the development of NEC [[2], [3], [4], [5]]. LPS, a component of gram-negative bacteria, is known to act as a trigger for NEC [30]. To replicate NEC conditions in vitro, FHC cells were subjected to LPS exposure. Additionally, a rat NEC model was created in vivo using hypoxia and formula feeding [28,31]. In this research, we discovered that reducing miR-155 decreased apoptosis, ferroptosis, inflammation, and oxidative stress in LPS-treated FHC cells through the upregulation of SLC7A11. Furthermore, lowering miR-155–5p levels could mitigate intestinal damage in NEC rats by enhancing SLC7A11 expression. These findings demonstrated that inhibiting miR-155–5p could alleviate experimental NEC by promoting SLC7A11.

MiR-155 is crucial in diseases associated with inflammation [32,33]. De Smet et al. demonstrated that reducing miR-155 could improve pulmonary inflammation in mice caused by cigarette smoke [34]. Wan et al. found that inhibition of miR-155 could induce pancreatic injury and inflammation in mice with acute pancreatitis [35]. Cao et al. discovered that a miR-155 blocker could reduce inflammation and restore intestinal barrier function in sepsis-afflicted mice by inhibiting NF-κB signalling [36], indicating that miR-155 might be a proinflammatory mediator in some inflammation-related diseases. Additionally, miR-155 contributes to inflammation in intestinal disorders like ulcerative colitis and inflammatory bowel disease [21,37]. In this research, we observed that reducing miR-155 levels could mitigate the inflammatory response in LPS-treated FHC cells and NEC rat intestinal tissues, evidenced by a corresponding decrease in p-IκBα, p65, IL-6, and TNF-α. Overall, targeting miR-155–5p improved intestinal damage in NEC by inhibiting inflammation.

Moreover, the analysis of information from the TargetScan and miRwalk repositories revealed that SLC7A11 is directly regulated by miR-155–5p, a molecule implicated in the process of ferroptosis [38]. The suppression of the cellular antioxidant defense mechanism triggers ferroptosis, which ultimately results in the accumulation of lipid peroxidation [39,40]. Additionally, ferroptosis is characterized by disrupted GPX4/GSH redox defence and the formation of detrimental ROS [39,40]. SLC7A11 has been found to participate in GSH biosynthesis [39]. Meanwhile, GPX4 could utilize GSH to detoxify lipid peroxidation [41]. Mitochondrial ROS production and the dysfunction of GPX4/GSH redox defence both cause lipid peroxidation, thereby triggering ferroptosis [39]. Ferroptosis is also characterized by the accumulation of intracellular iron. FTH1 has the activity of iron oxidase, which can catalyse the conversion of ferrous iron into ferric iron ions, thereby reducing the content of free iron and maintaining intracellular iron homeostasis [42]. In addition, COX-2 and ACSL4 are key enzymes involved in lipid peroxidation and have been shown to be increased in ferroptotic cells [43]. In this investigation, we observed diminished levels of SLC7A11, FTH1, GPX4, and GSH, along with elevated ROS generation in LPS-treated FHC cells and NEC rat intestinal tissues, however, the miR-155 inhibitor significantly reversed these effects. In addition, the miR-155 inhibitor markedly inhibited the Fe^2+^ level and reduced COX-2 and ACSL4 in LPS-treated FHC cells. Lots of studies have reported that inhibition of ferroptosis alleviates intestinal injury in multiple intestinal diseases [44,45]. In summary, the inhibition of miR-155–5p enhanced intestinal repair in MECs by decreasing ferroptosis.

This study discovered that inhibiting miR-155–5p could prevent cell ferroptosis in NEC. However, the connections between other differentially expressed miRNAs (such as miR-124–5p and miR-132–3p) and cell ferroptosis in NEC are still largely unclear. Thus, further investigations are essential to delve into the possible molecular pathways underlying the functions of miR-124–5p and miR-132–3p in necrotizing enterocolitis.

## 5 Conclusion

The visual representation of this study is presented in Supplementary Fig. 2. Overall, lower levels of miR-155–5p alleviated intestinal damage in NEC by suppressing ferroptosis and inflammation. These results could offer theoretical backing for the development of new therapies for NEC.

**Table:** Abbreviations

Abbreviation	Full name
GSH	Glutathione
SOD	Superoxide dismutase
CAT	Catalase
MDA	Malondialdehyde
ROS	Reactive oxygen species
MMP	Mitochondrial membrane potential
SLC7A11	Solute carrier family 7 member 11
FTH1	Ferritin heavy chain 1
GPX4	Glutathione peroxidase 4
COX-2	Cyclooxygenase-2
ACSL4	Acyl-CoA synthetase long-chain family member 4

## Data availability statement

The data that support the findings in this work are available from the corresponding author upon reasonable request.

## Ethical approval

This study was approved by the Ethics Committee children's Hospital of the Affiliated Wuxi Children's Hospital of Nanjing Medical University (Approval number WXCH2021-09-012), and all methods were implemented in accordance with the guidelines and regulations of animal ethics standards.

## CRediT authorship contribution statement

**Le Zhang:** Writing – original draft, Project administration, Data curation. **Weilai Jin:** Writing – original draft, Validation, Methodology, Investigation, Formal analysis. **Mengyuan Hu:** Validation, Investigation, Formal analysis. **Yinglin Su:** Software, Investigation, Formal analysis. **Yiting Zhang:** Software, Formal analysis. **Fuqiang Yuan:** Software. **Yuanyuan Fang:** Formal analysis. **Zhengying Li:** Software. **Yawen Li:** Validation, Supervision, Data curation. **Chaozhi Bu:** Writing – review & editing, Supervision, Project administration, Conceptualization. **Wenhao Zhou:** Conceptualization, Funding acquisition, Methodology, Supervision, Writing – review & editing.

## Declaration of competing interest

The authors declare that there are no conflicts of interest.

## Figures and Tables

**figs1:**
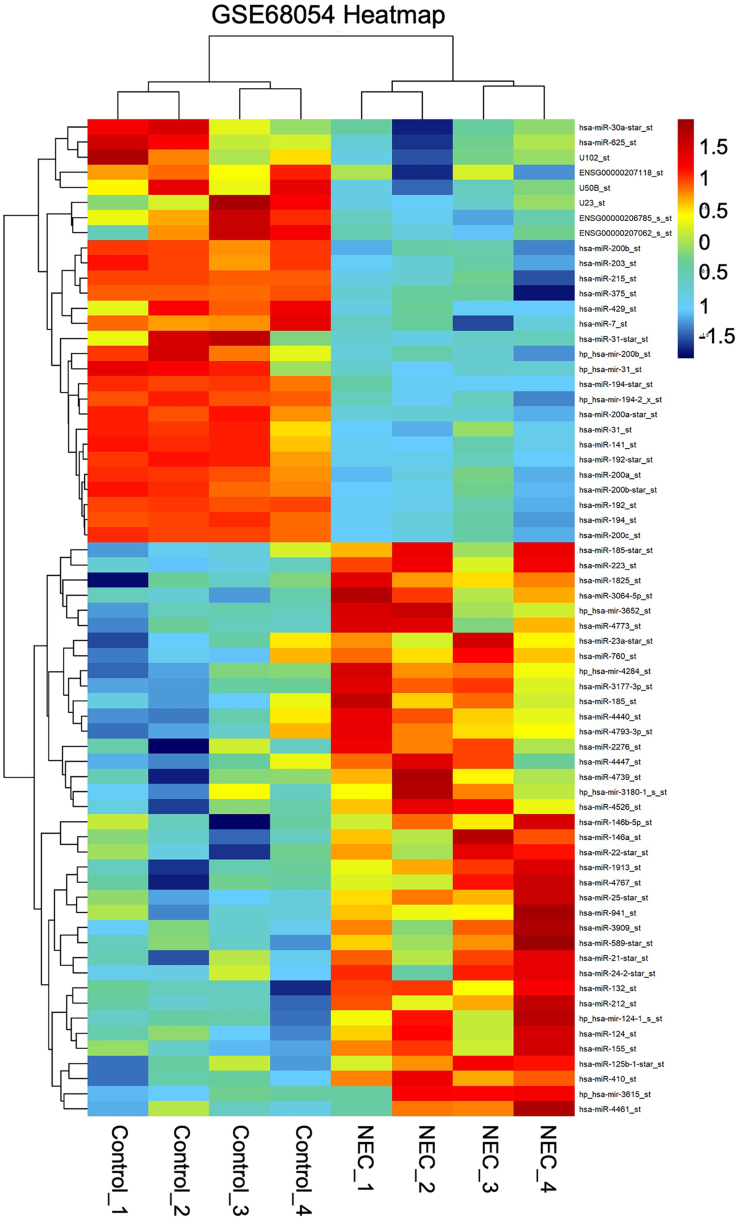


**figs2:**